# Application of Intra-articular Corticosteroid Injection in Juvenile Idiopathic Arthritis

**DOI:** 10.3389/fped.2022.822009

**Published:** 2022-03-29

**Authors:** Sha Li, Wei Zhang, Yan Lin

**Affiliations:** ^1^Department of Rheumatology, The Affiliated Women's and Children's Hospital, School of Medicine UESTC, Chengdu Women and Children's Central Hospital, Chengdu, China; ^2^Department of Outpatient, The Affiliated Women's and Children's Hospital, School of Medicine UESTC, Chengdu Women and Children's Central Hospital, Chengdu, China

**Keywords:** corticosteroid, intra-articular injection, juvenile idiopathic arthritis, juvenile, application

## Abstract

Juvenile idiopathic arthritis (JIA) is one of the common rheumatic diseases in pediatrics. Persistent synovitis and joint pain cause reduced range of motion, deformity and gait interruption, which are important reasons for children's disability and a decline in their quality of life. Rheumatology experts have explored good treatment strategies, among which intra-articular corticosteroid injections (IACIs) targeting joints can greatly reduce these systemic adverse reactions while still obtaining local anti-inflammatory effects. Local inhibition of synovitis by the use of steroid hormones in a joint cavity can avoid or reduce adverse reactions of systemic therapy, prevent or treat leg length variance and joint contracture, solve Baker's cyst, improve tenosynovitis, promote physical therapy and rehabilitation, make gait change smoothly, relieve pain, and restore joint function. Given the importance of IACIs in treating JIA, this paper reviewed the case selection, drug injection, dose selection, current anesthesia and injection techniques, the efficacy, recurrence, and influencing factors of IACIs, the management of physiotherapy intervention post-injection, the application of ultrasound guidance and the safety and complications of IACIs in children with JIA. This study aims to guide the use of IACIs for the best approach throughout the review.

## Introduction

Intra-articular corticosteroid injections (IACIs) are mainly applicable to chronic rheumatoid arthritis and osteoarthritis of the knee (1). Studies have shown that IACIs apply to any subcategory of juvenile idiopathic arthritis (JIA), especially to JIA with few joints (1). It can be used as initial therapy, alone or as an emergency therapy when non-steroidal anti-inflammatory drugs are ineffective (2). Also, IACIs are one of the most common treatments for oligoarticular JIA (1, 3, 4). Many pediatric rheumatologists regard IACIs as their first choice in the treatment of oligoarticular JIA (4–6). Clinical guidelines of the American College of Rheumatology recommend intra-articular glucocorticoids (IAGs) as the first-line treatment for JIA with few joints (7), and IACIs can also play a role in treating JIA with multiple joints. At present, in medical practice, the use of IACIs for multiple joints has increased. With multi-joint involvement, IACIs can only apply to a few intractable joints that do not respond to intensive systemic therapy (8). Scott et al. suggested that children with multi-joint JIA should undergo IACI several times to induce disease relief and start using second-line or biological agents (9). Administration of IACIs for several problematic joints is a substitute for systemic glucocorticoids. While waiting for the comprehensive therapeutic effect of disease-modifying antirheumatic drugs or biological drugs, the use of IACIs can quickly control inflammatory symptoms, treat joint deformity and relieve pain. Multiple IACIs have the potential advantages of avoiding many side effects of systemic corticosteroids and selectively targeting inflamed joints. Standard nursing ethics means this field lacks many randomized controlled trials (RCTs) and meta-analysis cannot be conducted. High-quality RCTs and meta-analyses are necessary to provide information for evidence-based clinical practice. However, it is recognized that despite the lack of high-quality research, there are still some clinical practice guidelines to support the evidence of its use (10).

## Methodology

In this study, MEDLINE, PubMed, EMBASE, the web of science and Cochrane libraries were used for comprehensive literature retrieval. The inclusion criteria were: meta-analysis, RCT, cohort study, observational study, case series (prospective/retrospective), and its outcome variables evaluated the clinical case selection dose and efficacy, safety, adverse reactions and/or puncture technology, anesthesia technology, and radiologic effects of IAGs on patients.

### Selection of Drugs, Dosage, and Frequency

Depot corticosteroids are most suitable for intra-articular injection therapy. The solubility of these drugs is low, and they can continue to play a role at the injection site of the joint. The duration of efficacy is inversely proportional to the solubility of the drug; the lower the solubility, the longer the duration of efficacy. At present, commonly used drugs include methylprednisolone (MP), triamcinolone acetonide (TA), triamcinolone hexanoate (TH), where the solubility is MP > TA > TH (Table 1). For preparing IACIs, TH has a longer duration of action than other preparations (8, 11, 12). The research of Zulian et al. showed that when one joint was infused with a standard dose of TH, while the other was infused with a double dose of TA, a higher proportion of joints infiltrated by TH entered the remission stage for a longer time. It shows that the joint injected with TH is more likely to get relief than the joint injected with TA, and, even if TA is used at a higher dose, TH is still more effective than TA (11, 13). The studies by Eberhard et al. also suggest that TH has a better control effect on joint symptoms than TA (12, 14). Eberhard reported in 2004 that the recurrence time after joint injection with TH was 10.14 months, while with TA, it was 7.75 months (15).

**Table 1:** Commonly used intra-articular steroids and doses.

**Joint**	**TH**	**TA**	**MP**
Shoulder, knee, hip	1 mg/kg (max 40 mg)	2 mg/kg (max 80 mg)	
Elbow, ankle	0.75 mg/kg (max 30 mg)	1.5 mg/kg (max 60 mg)	
Wrist	0.5 mg/kg (max 20 mg)	1 mg/kg (max 40 mg)	
Small hand and foot joint			5–10 mg
Subtalar and intertarsal			20–40 mg

*TH, triamcinolone hexanoate; TA, triamcinolone acetonide; MP, methylprednisolone*.

Literature shows that, although triamcinolone is the best glucocorticoid for IACIs, more soluble glucocorticoids (such as MP) are recommended for use at the facet joints (1, 9) to avoid subcutaneous atrophy or insufficient pigmentation (1, 11). Doses of TH range from 0.25 to 1.5 mg/kg depending on the size of the children and the joints (for example, 0.25–0.5 mg/kg for the wrist; 1–1.5 mg/kg for knees or hips) (8, 9). For small joints, such as the metacarpal and interphalangeal joints, the dose range of MP is 5–10 mg (9). According to pharmacokinetic studies, if the biological effect of TA at a double dose is equivalent to that of TH (16), it is usually re-injected when synovitis recurs. Although there is no limit to the number of joints that may require injections in each operation, systemic effects are inevitable when many joints are injected. The author thinks the maximum recommended number for each IACI treatment is three joints (1).

### Sedative Anesthesia Techniques

Before performing joint cavity punctures, the main concern is the anxiety and pain related to the operation. Many factors interact with and affect children's painful experiences, including environmental, cognitive, behavioral and biological factors. Children's anxiety before undergoing painful medical procedures is directly related to the pain experienced. Nieto-González and Monteagudo strongly recommends the use of local anesthesia before or during surgery and the use of conscious sedatives in specific cases for evaluation (1). The purpose of distraction therapy is to divert patients' attention from the source of pain. These methods have been proven to reduce their anxiety and improve physiological parameters such as blood pressure and tachycardia. The existence of well-trained medical clowns greatly reduces children's preoperative anxiety (17, 18). Because children with JIA often need repeated injections throughout their illness, extra care should be taken to make the injection process as painless and stress-free as possible, especially for the first injection. Therefore, painless methods can increase patients' comfort and promote the successful placement of steroids in joints.

To relieve pain, IACIs treatment can be performed under local anesthesia, conscious sedation or general anesthesia. No matter which technique is used, sufficient anesthesia or relaxation must ensure accurate injection. Local facilities and personal preferences and experience may become important factors in technology selection. Although IACIs are often used in the treatment of JIA, there are few studies on the efficacy of different anesthetics and sedation methods for this operation (19), and there is more evidence to use conscious sedation or local anesthetics to relieve pain with a joint puncture (20–24). Pastore et al. suggested that surgical sedation before injecting corticosteroids into joints in JIA should be the nursing standard (23). It has been suggested using a nitrous oxide (N_2_O) and oxygen inhalation mixture to help with children's painful operations, including IACIs. This procedure has the advantages of short-term hospitalization and avoiding the risks caused by intravenous sedation or general anesthesia (25–28). Three subsequent international studies (outside the United States) also described the use of N_2_O and concluded that it could provide safe and effective IACIs analgesia for children. Using N_2_O with IACIs and the active participation of medical clowns provides distraction techniques that have been shown to further reduce pain and stress (29).

Nieto-González and Monteagudo strongly recommends the use of local anesthetics, and conscious sedation is also an option for patients under 6 years old or who need multiple joint injections (1). Elitsur believes that a minimum sedation and anesthesia scheme (including N_2_O, intranasal fentanyl and ondansetron, local anesthetic, acetaminophen, ibuprofen, and distraction techniques) can provide safe and effective analgesics for some adolescent patients with idiopathic arthritis and pediatric arthritis. The use of IACIs provides a low-cost alternative to general anesthesia (30). Weiss et al. conducted an observational study to compare the use of local anesthetics (lidocaine cream or lidocaine iontophoresis) with subcutaneous lidocaine. They found that three times during the observed operations (before and after infiltration and after IACI), there was no difference in pain between patients who received a subcutaneous injection of lidocaine and lidocaine ointment and patients who only received local anesthesia with lidocaine ointment (22).

### Intra-Articular Injection Techniques

During intra-articular injections, most clinicians usually draw as much synovial fluid as possible. Although this practice has never been controlled in JIA, it seems logical and can immediately relieve symptoms (5). Aspiration of synovial fluid before corticosteroid injection is also a method to improve accuracy. We suggest to first draw as much synovial fluid as possible before IACIs to relieve symptoms immediately, which can improve the therapeutic effect because it requires a smaller volume of corticosteroids. Second, to increase the chances of success of IACIs treatment and minimize the risk of local side effects (i.e., subcutaneous atrophy), the needle must be placed correctly in the joint space. There is no consensus on such issues as flushing the needle track with saline or local anesthetic, mixing corticosteroid preparation with local anesthetic and “pulse” injection, especially in small dose injections (5).

### Curative Effect

Most literature has confirmed the efficacy of IACIs in the treatment of JIA. According to a research report, up to 82% of patients with JIA have achieved complete remission after injection with TH, resulting in 61% of patients stopping all oral medication (31). According to the study by Hashkes, 70% of patients with oligoarthritis have no disease reactivation for at least 1 year, while 40% have none for more than 2 years (4). Verma's study found that when IACI of TA was used as a day care procedure to treat JIA, 53.4% of children had no pain or limp or required the use of non-steroidal anti-inflammatory drugs, and the functional score improved at 12 weeks (32). Padeh thought treatment with IACIs could promote the discontinuation of oral medication, correct joint contracture, solve Baker's cyst and improve tenosynovitis (33). Decroix proved that isokinetic and isometric muscle strength measurements are reliable and effective methods for evaluating children's muscle strength (34), and isokinetic and isometric muscle strength have been proven to decrease in patients with JIA (35). Utilizing IACIs results in an early recovery and can reduce or even stop the use of systemic drugs (36, 37). Carmangotte's research suggests that IACIs can control disease activities, induce synovitis to subside, reduce joint and limb deformities, improve functioning, relieve pain, and reduce the need for more toxic treatments (38). Lanni et al. found that treatment with IACIs in a considerable number of patients injected in single or multiple joints can induce sustained relief of synovitis and has good safety (39). McKay thought that the improvement of the strength of the extensor and flexor muscles was considered to be of clinical significance after active treatment of inflamed knee joints with IACIs (40). In 2010, Scott re-evaluated IACIs for children with JIA and confirmed that IACIs are a safe, rapid, and effective treatment method (9). In 2014, Jennings' systematic evaluation found that IACIs have beneficial effects. However, there is contradictory and uncertain evidence about the curative effect of lower limb IACIs on JIA (41). In 2020, the results of a systematic evaluation and meta-analysis by Antonarakis showed IACIs could relieve pain and improve the mouth opening ability in children with a temporomandibular joint issue diagnosed as JIA (42).

### Recurrence and Influencing Factors

Regarding the influencing factors of recurrence after IACIs, different joints, disease subtypes, disease time, sex, inflammation index, whether the antinuclear antibody is positive, whether methotrexate (MTX) is used at the same time and whether uveitis is accompanied result in different curative effects. Breit's research showed that the median duration of curative effect varies with joints and disease subtypes. Patients with oligoarthritis before the age of five had the longest median duration, while patients with systemic arthritis had the shortest duration (43). Ravelli et al. found that response rates and duration for knees were longer than for buttocks, and other predictors of favorable response included shorter duration and younger age at IACIs, males and a higher erythrocyte sedimentation rate (ESR) (44). The risk of synovitis flare was higher in patients who had positive C-reactive protein (CRP), negative antinuclear antibodies (ANA) and were injected in the ankle (39). Ravelli et al. analyzed the response of TA in 94 cases (81 cases with few joints) treated on both knees by logistic regression analysis, in which the response was defined as the complete remission of synovitis at 6 months, and ESR was the only predictable favorable outcome index (44). Studies have shown that more than half of the joints showed a good response to IACIs, and those with uveitis responded well to IACIs during the disease. Knees, wrists, and elbows were the most effective joints for IACIs (45).

In a study involving 220 patients with JIA with 1,096 IACIs, the strongest predictor of synovitis recurrence after the IACIs was positive for CRP, negative ANA, lack of simultaneous administration of MTX, and the occurrence of multiple joints (as opposed to fewer joints) diseases (46). Ravelli et al. recently solved the problem of whether giving MTX at the same time would increase the frequency and duration of joint disease relief in children with oligoarticular JIA treated by IACIs in a prospective, randomized and open trial. This multicentre RCT in Italy compared IACIs with IACIs and oral MTX in children with oligoarticular JIA. Although the difference between the two treatment groups was not significant in the analysis of the main outcome (remission of arthritis symptoms of all injected joints in 12 months), the multivariate analysis and Cox proportional hazards model showed simultaneous MTX could prolong and enhance the effectiveness of IACIs to a lesser extent. Safety assessments showed no significant increase in severe toxicity (47).

### Physical Therapy Intervention After Injecting

At present, the benefit of specific physical therapy in patients with JIA after IACIs is still inconclusive. In 2003, Cleary et al. reported that the problems of rest, splint and physical therapy post-injection were still controversial and had not been studied in children's controlled trials. In adults, the evidence is confusing and inconclusive, according to Cleary. There are persistent and significant differences in the improvement of clinical and laboratory functions among patients who have been on strict bed rest for 24 h post-injection, though some studies suggest that strict bed rest is beneficial but unnecessary for IACIs (5). McKay's research in 2013 showed that the improvement in the extensor strength of treated knee joints was insufficient to ensure equal strength with unaffected limbs. Therefore, after injecting corticosteroids into the joint cavity, an exercise program may be needed, and confirmation of persisting deficits in knee extensor strength indicates that specific intensive exercise or physical therapy should be carried out after IACIs to resolve the strength deficiency. This is not only to improve strength but also to improve function and solve biomechanical abnormalities in gait (40). In 2019, Elaine et al. compared the group that was not splinted with the group that was splinted after IACI of the knee and concluded that the risk of recurrent arthritis was not statistically significant (48). Therefore, the physical therapy intervention after intra-articular injection of steroid hormones needs further study.

### The Value of Ultrasound Guidance

In the pediatric population, there is not enough scientific evidence to recommend the use of ultrasound scanning guidance in all IACI procedures (49). There are many descriptions of its use in different hospitals, but they do not compare the efficacy of operations performed with or without ultrasound scanning guidance (50, 51). Because the size of the facet joints and subcutaneous fat masks the bony markers, intra-articular puncture without ultrasound guidance is sometimes difficult. Therefore, in recent years, musculoskeletal ultrasound (MSUS) has been used to accurately place the needle tip in different affected anatomical structures in order to maximize the therapeutic effect. The literature reported the effectiveness of MSUS-guided injection of clinically inaccessible joints (49, 52). Magni-Manzoni believes MSUS shows great hope in the evaluation and management of children's JIA, which can be further studied (53).

### Safety and Complications

#### Security

There are many studies on the safety of IACIs, and most of them think IACIs are a safe and effective treatment for patients with JIA (2, 39, 54, 55). Marti's studies have shown that they are especially suitable for joints of the upper limbs and knees and in combination with methotrexate. Although side effects are very common, they are usually mild and do not require intervention (11).

#### Complications

Although systemic and local adverse reactions have also been reported, they are significantly less than those of systemic medication, with an incidence of only 2% reported in the literature (56).

##### Subcutaneous Atrophy

Local atrophy (skin or subcutaneous) is the most common adverse event (8), which is caused by the extravasation of drugs injected into the joint cavity. In most patients, subcutaneous atrophy may subside over time, but it will persist in some patients. Strict injection techniques and ensuring injection accuracy (including radiography) can minimize the risk of subcutaneous atrophy (43). The use of TH, a corticosteroid preparation with higher efficacy and duration of action, has the greatest risk of subcutaneous atrophy, and the risk is higher in smaller joints, so corticosteroids with a stronger solubility (betamethasone or MP) are better choices in small or superficial joints to avoid subcutaneous atrophy or insufficient pigmentation (1, 9).

##### The Systemic Effects of Cortisol

Although systemic absorption of corticosteroids may lead to obvious adrenal suppression and transient clinical manifestations from slight cosmetic changes to obvious Cushing's syndrome, it has nothing to do with long-term adverse reactions and is transient. The use of IACIs may cause systemic adverse events, such as Cushingoid habitus, transient adrenal suppression, acne-like rash, and insomnia (8, 56–58).

##### Others

Another known complication of IACIs is the development of periarticular calcifications. The majority of these abnormalities are asymptomatic and are detected coincidentally on radiological follow-up. Nicolau syndrome has even been reported after IACIs (59). Abnormal adverse reactions, including joint erythema, and pain after injection, were also encountered. Some researchers think this is the result of synovitis caused by crystals, in which the phagocytosis of steroid crystals in the joints leads to the release of inflammatory mediators. It usually subsides spontaneously within a few days or after local ice compresses.

## Conclusions

The use of IACIs for JIA is a safe and effective method for treating synovitis. It can be performed under local anesthesia with or without conscious sedation or under general anesthesia, and is an important treatment choice for children with JIA. This treatment, the so-called “bridge” effect, is an alternative to the use of systemic corticosteroids. However, due to the lack of controlled studies, the current position of IACI treatment in the management of children with JIA is still uncertain. Systematic, controlled and prospective research is needed to determine the best role of IACIs in children with JIA. In addition, it is necessary to support the training of pediatric rheumatologists in IACI technology and further explore the value of physiotherapy and ultrasound guidance.

## Author Contributions

SL and YL conceived the study and participated in its design and coordination. WZ helped to draft the manuscript. All authors have read and approved the final manuscript.

## Funding

This work was supported by 2020 Sichuan Maternal and Child Health Association Maternal and Child Medicine Science and Technology Innovation Project (Key Project) 2020ZD06, Chen Xiaoping Foundation for the Development of Science and Technology for Hubei Province CXPJJH121002-202149, 2020 Sichuan Health Research Project (Key Research Project) 20ZD019, and Chengdu High-Level Key Clinical Specialty Construction Project.

## Conflict of Interest

The authors declare that the research was conducted in the absence of any commercial or financial relationships that could be construed as a potential conflict of interest.

## Publisher's Note

All claims expressed in this article are solely those of the authors and do not necessarily represent those of their affiliated organizations, or those of the publisher, the editors and the reviewers. Any product that may be evaluated in this article, or claim that may be made by its manufacturer, is not guaranteed or endorsed by the publisher.
